# *Houttuynia cordata* Exhibits Anti-Inflammatory Activity Against Interleukin-1β-Induced Inflammation in Human Gingival Epithelial Cells: An In Vitro Study

**DOI:** 10.3390/dj13080360

**Published:** 2025-08-07

**Authors:** Ryo Kunimatsu, Sawako Ikeoka, Yuma Koizumi, Ayaka Odo, Izumi Tanabe, Yoshihito Kawashima, Akinori Kiso, Yoko Hashii, Yuji Tsuka, Kotaro Tanimoto

**Affiliations:** 1 Department of Orthodontics, Graduate School of Biochemical and Health Sciences, Hiroshima University, 1-2-3 Kasumi, Minami-ku, Hiroshima City 734-8554, Hiroshima, Japan; ryoukunimatu@hiroshima-u.ac.jp (R.K.); tsuka018@hiroshima-u.ac.jp (Y.T.); tkotaro@hiroshima-u.ac.jp (K.T.); 2 Research Center, Maruzen Pharmaceuticals Co., Ltd., 1089-8 Sagata Shinichi-cho, Fukuyama City 729-3102, Hiroshima, Japan; s-ikeoka@maruzenpcy.co.jp (S.I.); y-kawashima@maruzenpcy.co.jp (Y.K.); a-kiso@maruzenpcy.co.jp (A.K.); y-hashii@maruzenpcy.co.jp (Y.H.); 3 Department of Orthodontics, Division of Oral Health and Development, Hiroshima University Hospital, 1-2-3 Kasumi, Minami-ku, Hiroshima City 734-8554, Hiroshima, Japan; ayakaodo@hiroshima-u.ac.jp

**Keywords:** *Houttuynia cordata*, gingival epithelial cells, inflammation

## Abstract

**Background/Objectives**: Periodontitis is a chronic infectious inflammatory disorder that affects the supporting structures of the teeth. The gingival epithelium plays a crucial role as a physical and immunological barrier, producing pro-inflammatory cytokines in response to microbial pathogens. Modulation of gingival epithelial function has been proposed as a therapeutic strategy to prevent the progression of periodontal disease. *Houttuynia cordata*, a perennial herb traditionally used in Asian medicine, is recognized for its anti-inflammatory properties, with documented benefits in the cardiovascular, respiratory, and gastrointestinal systems. However, its potential therapeutic role in oral pathologies, such as periodontitis, remains underexplored. This study aimed to investigate the anti-inflammatory effects of *H. cordata* extract on interleukin (IL)-1β-stimulated primary gingival keratinocytes (PGKs) subjected to IL-1β-induced inflammatory stress, simulating the conditions encountered during orthodontic treatment. **Methods**: Inflammation was induced in PGKs using IL-1β, and the impact of *H. cordata* extract pretreatment was assessed using quantitative real-time reverse transcription polymerase chain reaction, enzyme-linked immunosorbent assay, and immunoblotting. **Results**: *H. cordata* extract significantly downregulated the mRNA and protein expression levels of tumor necrosis factor-alpha, IL-8, and intercellular adhesion molecule-1 in IL-1β-stimulated PGKs without inducing cytotoxicity. **Conclusions**: These findings suggest that *H. cordata* holds promise as a preventive agent against periodontitis by attenuating inflammatory responses in gingival epithelial tissues. We believe that our findings will inform the development of prophylactic interventions to reduce periodontitis risk in patients undergoing orthodontic therapy.

## 1. Introduction

*Houttuynia cordata* is a perennial herbaceous species that thrives in hydrophilic soils and temperate climates. It has been used for millennia in East Asian traditional medicine for both nutritional and therapeutic purposes [1,2,3,4]. Previous pharmacological studies have shown that *H. cordata* and its bioactive compounds confer cardioprotective [5], pulmonoprotective [6,7], and gastroprotective effects [8,9], which are largely attributable to their anti-inflammatory mechanisms of action. Additionally, research has highlighted its broad-spectrum pharmacological activities, including anticancer, antibacterial, and antiviral properties, which underscore its potential as a versatile therapeutic agent [1,3].

Given its extensive range of bioactivities, it is essential to explore the therapeutic potential of *H. cordata* for managing oral pathologies. It has been shown that extracts of *H. cordata* can suppress the expression of inflammation-related genes in human gingival epithelial cells stimulated by *Aggregatibacter actinomycetemcomitans*, but there has been insufficient research on the usefulness of *H. cordata* in the oral cavity [10]. Periodontitis, a prevalent oral inflammatory condition, is an infection-driven inflammatory disease initiated by periodontopathogenic bacteria. These bacteria proliferate in dental biofilms, which accumulate owing to suboptimal oral hygiene. A complex polymicrobial biofilm forms on the tooth surface, utilizing plaque as a substrate. Virulence factors produced by the biofilm infiltrate the gingival epithelium, provoking an inflammatory and immune cascade that activates host defense responses. This process culminates in the release of pro-inflammatory mediators, which contribute to extracellular matrix degradation and alveolar bone resorption [11]. Additionally, orthodontic appliance use has been recognized as a modifiable risk factor for periodontitis [12].

Orthodontic interventions are known to enhance patients’ oral health-related quality of life [13,14]; however, the presence of orthodontic appliances can complicate oral hygiene maintenance, disrupt the oral microbiota [15,16], and elevate the risk of periodontal disease [17,18]. Therefore, it is critical to develop effective prophylactic strategies to mitigate the risk of periodontitis during orthodontic treatment.

One of the key pro-inflammatory cytokines that is significantly upregulated in periodontal tissues during the initial stages of orthodontic therapy is interleukin (IL)-1β [19]. IL-1β enhances osteoclastogenesis and accelerates bone resorption by promoting the expression of matrix metalloproteinases in various cell types. Moreover, IL-1β is a pivotal mediator in the pathogenesis of periodontitis, with studies indicating its role in exacerbating periodontal inflammation and its reduction following periodontal therapy [20,21,22,23]. Consequently, attenuating the activity of inflammatory mediators in an IL-1β-rich microenvironment may be an effective therapeutic approach to prevent tissue degradation and alveolar bone loss associated with periodontitis, particularly during orthodontic treatment. Therefore, in this study, we hypothesized that *H. cordata*, which has shown inhibitory effects on inflammation-related genes in human gingival cells, might also be effective against inflammation induced by IL-1β. To date, no studies have investigated the anti-inflammatory potential of *H. cordata* in the context of IL-1β-induced periodontal inflammation.

This study aimed to investigate the anti-inflammatory effects of *H. cordata* on primary gingival keratinocytes (PGKs) subjected to IL-1β-induced inflammatory stress, simulating the conditions encountered during orthodontic treatment. We believe that our findings will inform the development of prophylactic interventions to reduce periodontitis risk in patients undergoing orthodontic therapy.

## 2. Materials and Methods

### 2.1. Preparation of H. cordata Extract

*H. cordata* extract (HOUTTUYNIA EXTRACT BG-3) was obtained from Maruzen Pharmaceuticals Co., Ltd. (Hiroshima, Japan). A freeze-dried product of the extract was dissolved in 50% dimethyl sulfoxide to a stock concentration of 5 mg/mL and subsequently diluted in the appropriate cell culture medium to achieve working concentrations for the experimental assays.

### 2.2. Cell Culture

Primary gingival keratinocytes (PGKs; PCS-200-014, ATCC, Manassas, VA, USA) were cultured in 75 cm^2^ tissue culture flasks (Nunc, 156499, Thermo Fisher Scientific, Waltham, MA, USA) using Dermal Cell Basal Medium (DCBM; PCS-200-030, ATCC) supplemented with a Keratinocyte Growth Kit (KGK; PCS-200-040, ATCC). The cells were maintained under standard incubation conditions at 37 °C in a humidified atmosphere containing 5% CO_2_. Upon reaching confluence, the cells were harvested using Trypsin-EDTA for Primary Cells (PCS-999-003, ATCC), neutralized with Trypsin Neutralizing Solution (PCS-999-004, ATCC), and subcultured in experimental plates.

### 2.3. Cell Proliferation Assay

Precultured PGKs were seeded into type 1 collagen-coated 96-well plates (4860-010, IWAKI, Tokyo, Japan) at a density of 2000 cells/well and incubated overnight. After incubation, the medium was replaced with DCBM lacking KGK, and the cells were reincubated for 3 h. Subsequently, based on a previous report [10], the medium was replaced with fresh DCBM containing varying concentrations of *H. cordata* extract, followed by a 22-h incubation. Cell proliferation was assessed using the Cell Proliferation ELISA Bromodeoxyuridine (BrdU) kit (1647229; Roche, Basel, Switzerland), following the manufacturer’s instructions. The absorbance values were measured using a microplate reader (Thermo Fisher Scientific Inc., Waltham, MA, USA).

### 2.4. RNA Isolation

PGKs cultured in DCBM supplemented with KGK were seeded into 6-well plates (Falcon, 353046, Falmouth, UK) at a density of 2.0 × 10^5^ cells/well and incubated for 72 h. The medium was then replaced with DCBM containing KGK without hydrocortisone hemisuccinate, and the cells were pretreated with varying concentrations of *H. cordata* extract for 3 h. Following this, cells were washed with fresh medium and exposed to Recombinant Human IL-1β (200-01B, PEPROTECH, Cranbury, NJ, USA) at a final concentration of 1 ng/mL for an additional 3 h. Total RNA was extracted using ISOGEN II (311-07361, NIPPON GENE, Osaka, Japan), according to the manufacturer’s instructions. RNA concentration was determined by measuring the absorbance at 260 nm using a microplate reader (Thermo Fisher Scientific Inc.), and the concentration was normalized to 200 ng/µL.

### 2.5. Reverse Transcription Polymerase Chain Reaction (RT-PCR)

Total RNA was reverse-transcribed into complementary DNA (cDNA) using PrimeScript™ RT Master Mix (Perfect Real Time) (RR036A, TaKaRa Bio Inc., Shiga, Japan). Quantitative RT-PCR was performed using a Thermal Cycler Dice^®^ Real-Time System III (TaKaRa Bio Inc.). The resulting cDNA was used to quantify the mRNA expression levels of tumor necrosis factor-alpha (TNF-α), IL-8, intercellular adhesion molecule-1 (ICAM-1), and glyceraldehyde-3-phosphate dehydrogenase (GAPDH), which served as the internal control. mRNA quantification was conducted using TB Green^®^ Fast qPCR Mix (RR430A, TaKaRa Bio Inc.) with gene-specific primer sets from TaKaRa Bio Inc. (TNF-α: HA252960, IL-8: HA032483, ICAM-1: HA334629, GAPDH: HA067812). The mRNA levels were normalized to those of GAPDH to determine the relative expression.

### 2.6. Cell Preparation for Protein Expression Analysis

PGKs, cultured in DCBM supplemented with KGK, were seeded at a density of 5.0 × 10^4^ cells/well in 24-well plates (Falcon; 353047) and incubated for 48 h. The culture medium was then replaced with DCBM containing KGK without hydrocortisone hemisuccinate, and the cells were pretreated with varying concentrations of *H. cordata* extract for 3 h. The cells were washed with fresh medium and exposed to 1 ng/mL of recombinant human IL-1β for an additional 48 h.

### 2.7. Enzyme-Linked Immunosorbent Assay (ELISA)

The culture supernatants were collected after 48 h, and TNF-α and IL-8 concentrations were measured using ELISA kits (DY210 for TNF-α and DY208 for IL-8; R&D Systems Inc., Minneapolis, MN, USA), following the manufacturer’s protocol.

### 2.8. Immunoblotting Analysis

Following the 48-h incubation, cells were rinsed with phosphate-buffered saline, and total protein was isolated using RIPA Lysis and Extraction Buffer (89900, Thermo Fisher Scientific Inc.) supplemented with Halt™ Protease Inhibitor Cocktail (100×) (87786, Thermo Fisher Scientific Inc.). Protein concentrations were quantified using a Pierce™ BCA Protein Assay Kit (23225, Thermo Fisher Scientific Inc.). Protein samples were denatured by heating at 95 °C for 3 min in sodium dodecyl sulfate–polyacrylamide gel electrophoresis (SDS-PAGE) Sample Buffer with Reducing Reagent (6×) (09499-14, Nacalai Tesque, Inc., Kyoto, Japan). A total of 5 μg of denatured protein per sample was separated by SDS-PAGE on Mini-PROTEAN TGX™ Gels (4–20%, 4561096, Bio-Rad Laboratories, Inc., Hercules, CA, USA) at a constant voltage of 200 V and 40 mA. Proteins were subsequently transferred to polyvinylidene fluoride membranes using the Trans-Blot^®^ Turbo™ Transfer System (1704150; Bio-Rad Laboratories, Inc.) and Trans-Blot^®^ Turbo™ RTA Transfer Kit (1704274; Bio-Rad Laboratories, Inc.).

After transfer, the membranes were blocked for 1 h at room temperature using EveryBlot Blocking Buffer (12010020; Bio-Rad Laboratories, Inc.). The blocked membranes were incubated with a primary antibody, rabbit anti-human ICAM-1 polyclonal antibody (15364-1-AP; Proteintech Group, Inc., Rosemont, IL, USA) diluted 1:2000 for 2 h at room temperature. After primary antibody incubation, the membranes were washed and incubated with secondary antibodies, either goat anti-rabbit IgG StarBright™ Blue 700 (12004162, Bio-Rad Laboratories, Inc.) or Anti-actin hFAB™ Rhodamine Antibody (12004164, Bio-Rad Laboratories, Inc.), both diluted 1:5000, for 1 h at room temperature. Protein signal detection was performed using the ChemiDoc™ Touch MP Imaging System (17001402J1PC, Bio-Rad Laboratories, Inc.) and quantified using Image Lab Software version 6.1 (12012931, Bio-Rad Laboratories, Inc.). ICAM-1 protein expression was quantified by normalizing its band intensity to the corresponding β-actin band intensity, which served as a loading control.

### 2.9. Statistical Analysis

All statistical analyses were performed using Dunnett’s test. For cytotoxicity tests, comparisons were made between the control group (without *H. cordata* treatment) and the *H. cordata*-treated group. For gene and protein expression analyses, the control group consisted of IL-1β-stimulated cells without *H. cordata* treatment and was compared with the *H. cordata*-treated groups at each concentration. Statistical significance was set at *p* < 0.05.

## 3. Results

In this study, we investigated the anti-inflammatory potential of *H. cordata* on PGKs by inducing an inflammatory response with IL-1β and assessed the prophylactic efficacy of *H. cordata* extract following pretreatment.

### 3.1. Cytotoxicity Assessment of H. cordata

**Figure 1:**
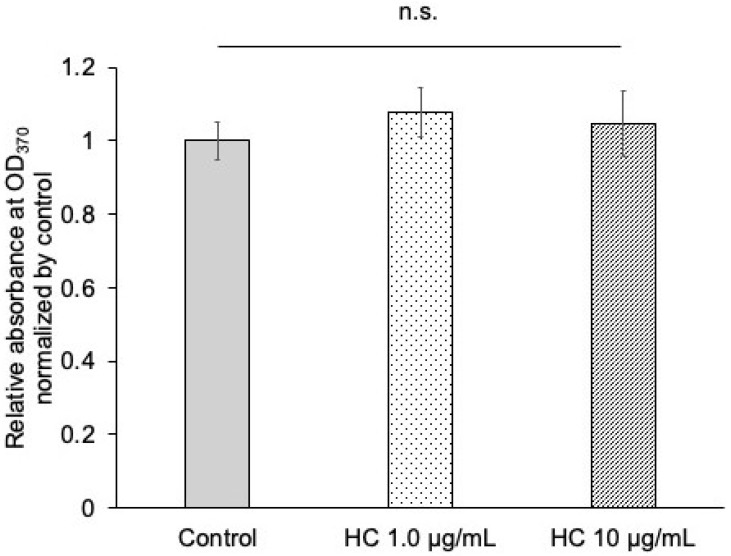
Effect of *H. cordata* on cell proliferation in primary gingival keratinocytes (PGKs). PGKs were treated with *H. cordata* at concentrations of 1 μg/mL and 10 μg/mL. Cell proliferation was assessed using the bromodeoxyuridine (BrdU) assay, with absorbance measured at 370 nm. No significant differences in cell proliferation were observed between the *H. cordata*-treated and control groups, indicating that *H. cordata* did not affect cell viability within the tested concentration range. n.s.; not significant.

The cytotoxicity of the *H. cordata* extract was assessed using a BrdU assay. Notably, incremental concentrations of *H. cordata* extract did not result in a significant reduction in cell viability (Figure 1), indicating that the extract was non-cytotoxic at concentrations between 1.0 µg/mL and 10 µg/mL. At a concentration of 100 µg/mL, *H. cordata* showed a significant decrease in absorbance in the BrdU assay.

### 3.2. mRNA Expression Analysis

**Figure 2:**
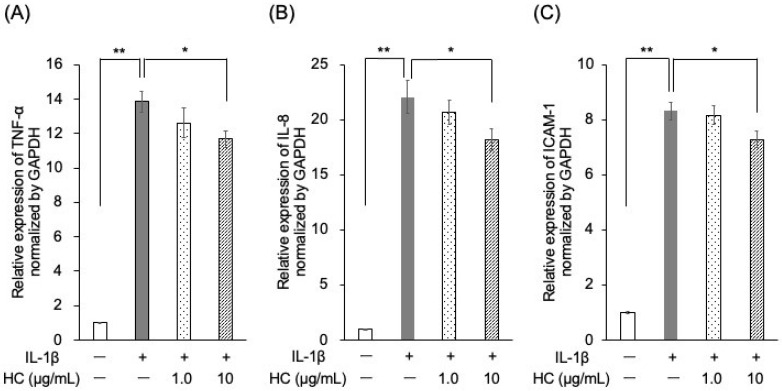
Effect of *H. cordata* on mRNA expression of inflammatory mediators induced by interleukin (IL)-1β in PGKs. PGKs were treated with IL-1β for 3 h after 3 h treatment with *H. cordata* (1 or 10 μg/mL), followed by RNA extraction. Reverse transcription polymerase chain reaction (RT-PCR) was performed to quantify the mRNA levels of (**A**) tumor necrosis factor-alpha (TNF-α), (**B**) IL-8, and (**C**) intercellular adhesion molecule-1 (ICAM-1), with glyceraldehyde-3-phosphate dehydrogenase (GAPDH) serving as an internal control (*n* = 4). The mRNA expression levels of TNF-α, IL-8, and ICAM-1 were significantly upregulated in cells treated with IL-1β. Treatment with 10 μg/mL of *H. cordata* significantly reduced the mRNA levels of these inflammatory mediators compared to the IL-1β-stimulated group, indicating an attenuation of IL-1β-induced inflammation. * *p* < 0.05, ** *p* < 0.01 compared to the control (Dunnett’s test).

Quantitative RT-PCR analysis revealed that the mRNA expression levels of TNF-α, IL-8, and ICAM-1 were significantly elevated in the IL-1β-stimulated control group compared to those in the unstimulated group (Figure 2A–C). Pretreatment with 10 μg/mL of *H. cordata* extract significantly reduced the mRNA expression levels of TNF-α, IL-8, and ICAM-1 compared to the IL-1β-stimulated control group.

### 3.3. Protein Expression Analysis

**Figure 3:**
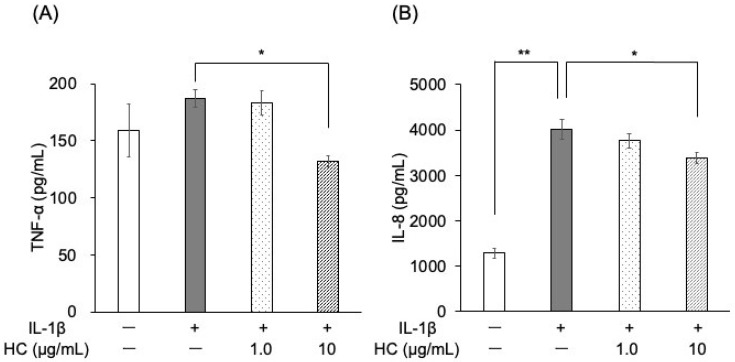
Effect of *H. cordata* on protein expression of TNF-α and IL-8 in PGKs. PGK culture supernatants were collected after 48 h of incubation with IL-1β following a 3 h treatment with *H. cordata*. Enzyme-linked immunosorbent assay (ELISA) was used to quantify the protein levels of (**A**) TNF-α and (**B**) IL-8 in the supernatants (*n* = 5). IL-1β stimulation resulted in significantly higher protein levels of TNF-α and IL-8 compared to the control. Pretreatment with 10 μg/mL of *H. cordata* significantly reduced these protein levels in IL-1β-stimulated cells. * *p* < 0.05, ** *p* < 0.01 compared to the control (Dunnett’s test).

**Figure 4:**
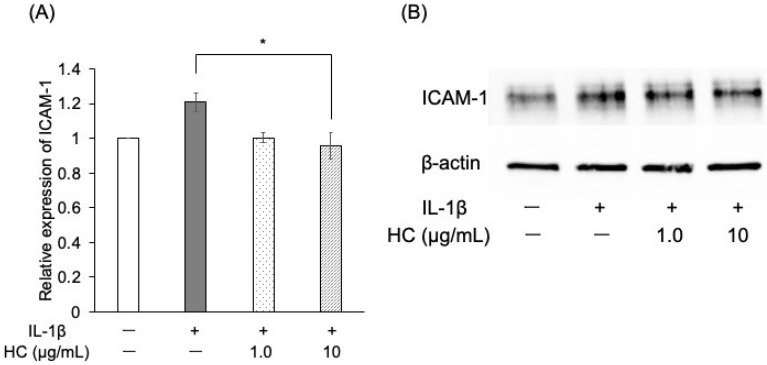
Effect of *H. cordata* on protein expression of ICAM-1 in PGKs. (**A**) PGK total protein was extracted after 48 h of incubation with IL-1β following a 3 h pretreatment with *H. cordata*. Immunoblotting was performed to assess ICAM-1 protein expression relative to β-actin. ICAM-1 protein expression was quantified (*n* = 3). ICAM-1 protein expression was significantly higher in IL-1β-stimulated cells than in the control group but decreased with 10 μg/mL *H. cordata*. (**B**) Representative bands of β-actin and ICAM-1 are shown. * *p* < 0.05, compared to the control (Dunnett’s test).

Forty-eight hours after IL-1β stimulation, the protein levels of IL-8 were significantly higher in the control group than in the unstimulated group (Figure 3B). TNF-α expression also showed an upward trend, although this trend was not statistically significant (Figure 3A). ELISA results demonstrated that pretreatment with *H. cordata* extract (10 μg/mL) significantly reduced the protein levels of both TNF-α and IL-8 compared to the IL-1β-stimulated control (Figure 3A,B). Immunoblotting analysis indicated that ICAM-1 protein expression was significantly lower in the *H. cordata*-treated group than in the control group at 48 h post-IL-1β stimulation (Figure 4A,B).

## 4. Discussion

The anti-inflammatory effects of several natural ingredients have been investigated in vitro. Chen et al. reported that *Gardenia jasminoides* suppresses the expression of cytokines (IL-1β, IL-6, and TNF-α) in ARPE-19 cells [24]. It has also been reported that compounds from *Angelica acutiloba* inhibit the production of IL-6 and TNFα in RAW264 cells in which inflammation was induced by LPS [25]. Morusin, a flavonoid from mulberry bark, inhibits the secretion of cytokines such as CCL5 and CCL17 in keratinocytes stimulated by TNF-α and IFN-γ [26]. Among these natural ingredients, *H. cordata* has been reported to have anti-inflammatory effects in many cases, and its efficacy in the oral cavity is expected.

Previous studies have demonstrated that *H. cordata* modulates innate immune mediators in the oral cavity without inducing cytotoxicity [27]. Additionally, it exhibits anti-biofilm properties against methicillin-resistant *Staphylococcus aureus*, *Fusobacterium nucleatum*, and *Candida albicans* and suppresses IL-8 and CCL20 production in *Porphyromonas gingivalis* lipopolysaccharide-stimulated oral keratinocytes, without cytotoxic effects [28]. Furthermore, *H. cordata* has been reported to attenuate gingival epithelial inflammation caused by *Aggregatibacter actinomycetemcomitans* and TNF-α by downregulating the expressions of matrix metalloproteinase 3, IL-8, IL-6, and ICAM-1 via the extracellular signal-regulated kinase signaling pathway [10]. Despite these findings, its therapeutic potential in oral diseases, including periodontitis, remains underexplored. To our knowledge, this study is the first to demonstrate that *H. cordata* can inhibit IL-1β-induced inflammation in PGKs, with pretreatment showing a significant anti-inflammatory effect. Our findings revealed that pretreatment with *H. cordata* extract significantly suppressed the inflammatory response in PGKs stimulated by IL-1β by reducing the mRNA and protein levels of TNF-α, IL-8, and ICAM-1. These results are consistent with those of previous reports, indicating that *H. cordata* modulates various inflammatory cytokines and adhesion molecules [29,30]. The addition of *H. cordata* before the rapid increase in IL-1β expression is expected to occur before the application of force in orthodontic treatment. Therefore, a condition similar to Periodontal Stage I (e.g., clinical attachment loss of ≤2 mm or periodontal pocket depth of ≤4 mm) in “The 2018 AAP/EFP Classification of Periodontal and Peri-implant Diseases” is expected. Further investigation is needed regarding the usefulness of *H. cordata* in conditions where IL-1β expression is already increased, not just in preventive administration as in this experiment.

TNF-α, a cytokine activated by nuclear factor-kappa B (NF-κB) [31,32,33], plays a crucial role in the immune response to periodontopathogenic bacterial infections and bone resorption [34,35,36]. It is also a well-known inducer of IL-8 production [37]. IL-8, an inflammatory chemokine, promotes leukocyte chemotaxis, DNA synthesis, and migration, while inhibiting cleaved caspase-3 in human gingival epithelial cells [38,39]. Elevated IL-8 expression is associated with periodontitis, and its expression is regulated by the NF-κB pathway, which is elevated in patients with chronic periodontitis [40,41,42]. ICAM-1, also regulated by NF-κB [42], acts as a ligand for lymphocyte function-associated antigen-1 on leukocytes, facilitating their migration and activation during periodontopathogenic bacterial infections [43].

Given the suppression of TNF-α, IL-8, and ICAM-1 observed in this study, the anti-inflammatory properties of *H. cordata* may be particularly beneficial in reducing the inflammatory response caused by IL-1β overexpression. The suppression of NF-κB expression may contribute to these outcomes, as previous research suggests that *H. cordata* inhibits NF-κB activation and nuclear translocation, reducing the mRNA levels of TNF-α, IL-6, and IL-8 in mast cells [44,45,46,47]. A similar mechanism may be operative in gingival epithelial cells, as indicated by our findings. Additionally, the reduced expression of these inflammatory markers supports the hypothesis that IL-1β-induced inflammation activates the NF-κB signaling pathway via a positive feedback loop, amplifying the inflammatory response [4]. *H. cordata* pretreatment may disrupt this feedback loop, thereby reducing inflammation.

Our results suggest that *H. cordata* is an effective anti-inflammatory agent for managing periodontal diseases. Incorporating *H. cordata* extract into mouthwashes and dentifrices could potentially prevent periodontitis associated with orthodontic treatment or serve as an alternative therapeutic approach for periodontal disease. However, this study had some limitations. First, while we utilized PGKs, periodontal tissues comprise various cell types, and further research is needed to evaluate the effects of *H. cordata* on other periodontal cell types, such as periodontal ligament cells. Second, detailed investigations of the signaling mechanisms involved are necessary. Finally, as this study was conducted in vitro, additional in vivo studies are required to determine the optimal dosage and administration methods for *H. cordata* extract, as well as its long-term safety and potential side effects. Furthermore, because the oral cavity is a complex environment that includes enzymatic degradation in saliva, an anaerobic environment, and bacterial biofilms, evaluation of the efficacy of *H. cordata* in in vivo models is also necessary. *H. cordata* is naturally derived and has been suggested to have various effects in humans, but its effects on the oral cavity and periodontal tissues in humans remain to be determined. Therefore, after validation in an animal in vivo study, it is necessary to conduct a high-quality blinded clinical study to provide detailed information on the effects of *H. cordata*.

## 5. Conclusions

The administration of *H. cordata* did not exhibit cytotoxicity towards PGKs. Pretreatment with *H. cordata* significantly attenuated the expression of TNF-α, IL-8, and ICAM-1 in PGKs in response to IL-1β-induced inflammation. These findings suggest that H. cordata may be an adjunct therapy for periodontitis by exerting anti-inflammatory effects on periodontal tissues. Further investigation is required to determine whether pretreatment with *H. cordata* also exerts an anti-inflammatory effect in in vivo models.

## Data Availability

The raw data supporting the conclusions of this article will be made available by the authors on request.

## Abbreviations

The following abbreviations are used in this manuscript:

CCL20	Chemokine ligand 20
DCBM	Dermal Cell Basal Medium
BrdU	Bromodeoxyuridine
ELISA	Enzyme-linked immunosorbent assay
GAPDH	Glyceraldehyde-3-phosphate dehydrogenase
ICAM	Intercellular adhesion molecule-1
IL	Interleukin
KGK	Keratinocyte Growth Kit
NF-κB	Nuclear factor-kappa B
PGK	Primary gingival keratinocyte
RT-PCR	Reverse transcription polymerase chain reaction
SDS-PAGE	Sulfate-polyacrylamide gel electrophoresis
TNF-α	Tumor necrosis factor-alpha
